# Association of Inflammatory Metabolic Activity of Psoas Muscle and Acute Myocardial Infarction: A Preliminary Observational Study with ^18^F-FDG PET/CT

**DOI:** 10.3390/diagnostics11030511

**Published:** 2021-03-13

**Authors:** Kisoo Pahk, Eung Ju Kim, Hyun Woo Kwon, Chanmin Joung, Hong Seog Seo, Sungeun Kim

**Affiliations:** 1 Department of Nuclear Medicine, Korea University Anam Hospital, Seoul 02841, Korea; kisu99@korea.ac.kr (K.P.); hnwoo00@gmail.com (H.W.K.); 2 Department of Cardiovascular Center, Korea University Guro Hospital, Seoul 08308, Korea; withnoel@empal.com; 3 Institute for Inflammation Control, Korea University, Seoul 02841, Korea; joungchanmin@korea.ac.kr

**Keywords:** coronary artery disease, acute myocardial infarction, psoas muscle, inflammation, atherosclerosis, positron-emission tomography

## Abstract

Inflamed skeletal muscle promotes chronic inflammation in atherosclerotic plaques, thereby contributing to the increased risk of coronary artery disease (CAD). In this study, we evaluated the metabolic activity of psoas muscle, using ^18^F-fluorodeoxyglucose (FDG) positron emission tomography/computed tomography (PET/CT), and its association with carotid artery inflammation and acute myocardial infarction (AMI). In total, 90 participants (32 AMI, 33 chronic stable angina (CSA), and 25 control) were enrolled in this prospective study. Metabolic activity of skeletal muscle (SM) was measured by using maximum standardized uptake value (SUVmax) of psoas muscle, and corresponding psoas muscle area (SM area) was also measured. Carotid artery inflammation was evaluated by using the target-to background ratio (TBR) of carotid artery. SM SUVmax was highest in AMI, intermediate in CSA, and lowest in control group. SM SUVmax was significantly correlated with carotid artery TBR and systemic inflammatory surrogate markers. Furthermore, SM SUVmax was independently associated with carotid artery TBR and showed better predictability than SM area for the prediction of AMI. Metabolic activity of psoas muscle assessed by ^18^F-FDG PET/CT was associated with coronary plaque vulnerability and synchronized with the carotid artery inflammation in the participants with CAD. Furthermore, it may also be useful to predict AMI.

## 1. Introduction

Cardiovascular disease (CVD) is the leading cause of death globally, and around 40% of these deaths are due to coronary artery disease (CAD) [1,2]. CAD is usually caused by atherosclerosis, which can further lead to plaque erosion or rupture that ultimately results in angina and/or acute myocardial infarction (AMI) [3].

Several previous studies have reported that increased inflammation in skeletal muscle (SM) could contribute to the upregulation of systemic inflammation, which may lead to the promotion of the chronic inflammatory process in atherosclerotic arterial lesions [4,5,6]. Inflamed SM secretes large numbers of pro-inflammatory cytokines, such as tumor necrosis factor-alpha (TNF-α) and monocyte chemotactic protein-1 (MCP-1) thereby promoting inflammatory cells infiltration into SM with predominant M1-polarized macrophages, which further contribute to insulin resistance with systemic inflammation and increase the risk of atherosclerotic plaque rupture [4,5,6]. Furthermore, SM has been accounted for 80% of insulin-stimulated glucose uptake in whole-body and has been regarded as the most important organ for glucose homeostasis [7,8,9]. Thus, inflamed SM could play a key role in the development of CVD.

^18^F-fluorodeoxyglucose positron emission tomography (^18^F-FDG PET/CT) is a non-invasive nuclear medicine imaging modality which uses ^18^F-FDG, a glucose analogue that metabolically accumulates in the cells, thereby reflecting cell glycolysis [10]. In current clinical practice, ^18^F-FDG PET/CT has been widely used to diagnose metastasis or monitor chemotherapy response in patients with tumor [11]. Recently, beyond its clinical application in oncology, it is possible to evaluate the inflammatory activity of specific tissue, particularly M1 macrophage activity, with ^18^F-FDG PET/CT [10]. This concept is strongly supported by previous studies which use ^18^F-FDG PET/CT imaging to evaluate atherosclerotic plaque vulnerability [10,11,12]. Furthermore, we found that ^18^F-FDG PET/CT could reflect the inflammatory activity of visceral adipose tissue in the patients with metabolic syndrome, which is a powerful risk factor for CVD [13]. Interestingly, similar to SM, inflamed visceral adipose tissue also induces insulin resistance and systemic inflammation, which collectively contributes to the escalated risk of CVD [14]. Thus, it is conceivable that ^18^F-FDG PET/CT might also reflect the inflammatory activity of SM.

Furthermore, recently, Kim et al. [15] reported that ^18^F-FDG uptake in the psoas muscle was increased in patients with metabolic syndrome, which is also a risk factor for CVD. Based on these findings, we hypothesized that the metabolic activity of psoas muscle evaluated by ^18^F-FDG PET/CT could also be associated with the severity of coronary plaque instability in CAD patients.

The aim of this prospective study was to investigate the relationship between the metabolic activity of psoas muscle assessed by ^18^F-FDG PET/CT and the severity of coronary plaque instability in the CAD participants, including AMI.

## 2. Materials and Methods

### 2.1. Study Participants

From June 2008 to March 2009, patients with newly diagnosed CAD, including AMI or chronic stable angina (CSA), were recruited in this prospective study. AMI was diagnosed as typical changes of biochemical cardiac injury markers along with ≥1 of the following: ischemic symptoms, electrocardiographic changes indicative of new ischemia, presence of pathological Q waves, or newly appeared regional wall motion abnormality, or imaging evidence of loss of myocardial viability [16]. CSA was defined as the development of stable angina symptoms for ≥6 months along with ≥50% luminal narrowing in at least one major coronary artery by angiographic assessment [17]. Control group consisted of consecutive age-matched participants who underwent a general health check up with ^18^F-FDG PET/CT, from June 2008 to March 2009. Exclusion criteria for control group were as follows: known history of previous CVD (myocardial infarction, unstable angina, stroke, and/or cardiovascular revascularization), greater than stage-1 hypertension (resting blood pressure ≥160/100 mmHg), cancers, uncontrolled diabetes mellitus (glycated hemoglobin > 9%), history of an inflammatory condition, severe renal or hepatic diseases, and/or taking any medications that could influence the systemic inflammatory condition within 6 months from this study. Lastly, a total of 90 participants were included and examined by ^18^F-FDG PET/CT. This study complied with the Declaration of Helsinki. All included participants provided written informed consent and The Institutional Review Board of Korea University Guro Hospital (Approval No. KUGH06114) approved the study design.

### 2.2. Anthropometric and Laboratory Measurements

Body mass index (BMI) was defined as weight/height squared (kg/m^2^), and waist circumference (WC) was measured from midway between the lowest rib and the iliac crest in the sitting position. All blood samples were collected after overnight fasting. The levels of lipid profiles and glycated hemoglobin were measured by a chemistry analyzer (Hitachi 747, Hitachi, Tokyo, Japan). The low-density lipoprotein cholesterol concentration was calculated based on the Friedewald formula [18]. The levels of high-sensitivity C-reactive protein (hsCRP) levels were measured by a chemiluminescence immunoassay (Beckman Coulter, Brea, CA, USA). Cardiac troponin-T and creatine kinase-MB fraction were calculated by an Elecsys 2010 analyzer (Roche Diagnostics, Indianapolis, IN, USA). Based on the instructions of manufacturer, the concentration of troponin-T > 0.1 ng/mL and creatine kinase-MB > 6.73 ng/mL in men or > 3.77 ng/mL in women were taken as cutoff values for the diagnosis of AMI.

### 2.3. ^18^F-FDG PET/CT Protocol

All participants underwent ^18^F-FDG PET/CT after overnight fasting. Participants with AMI, all of whom were treated with percutaneous coronary intervention successfully, took ^18^F-FDG PET/CT within 10 days after AMI onset when they were clinically stable. The image acquisition was started 60 min after injection of 5.29 MBq/kg ^18^F-FDG using a dedicated PET/CT scanner (GEMINI TF, Philips Medical Systems, Cleveland, OH, USA), which is composed of a lutetium-yttrium oxyorthosilicate full-ring time-of-flight capable PET and 16-slice helical CT. The scanning range covered the area from the skull base to the proximal thigh. The CT scan was performed first for attenuation correction and localization (4 mm thickness; 120 kVp; 50mA). Immediately after the CT scan, the PET scan was performed for 9 bed positions at 1 min per bed position (4.4 mm spatial resolution with 18 cm axial field of view). All the PET images were reconstructed by the iterative algorithm (three-dimensional row-action maximum likelihood algorithm), using the CT-based attenuation maps.

### 2.4. Image Analysis

Two experienced nuclear medicine physicians (Kisoo Pahk and Hyun Woo Kwon) analyzed the images using a commercially available workstation (Extended Brilliance Workspace version 3.5, Philips Healthcare, Eindhoven, Netherlands). To measure the carotid artery inflammation, regions of interest (ROIs) were located on the right carotid artery and jugular vein.

Standardized uptake value (SUV) was defined as the net ^18^F-FDG uptake in the ROI normalized by injected dose and body weight. It was calculated as follows:

SUV = ^18^F-FDG concentration in ROI (MBq/g)/Injected dose (MBq)/Total body weight (g) (1)

Next, the arterial target-to-background ratio (TBR) was calculated as averaged highest carotid artery SUV divided by averaged highest jugular vein SUV over all 8 consecutive slices, which started at the bifurcation and extended superiorly and inferiorly every 4 mm [17]. For the measurement of SM area and SM metabolic activity, psoas muscle area was identified on the CT images at the level of L4 spine [19]. ROIs were manually drawn on the right and left psoas muscles and the values of each area, and their corresponding maximum standardized uptake value (SUVmax) were acquired. SM area was defined as the mean value of right and left psoas muscle area. SM glucose uptake, which defined as SM SUVmax, was calculated as the mean value of right and left psoas muscle SUVmax. Increased metabolic activities of both spleen and bone marrow (BM) evaluated by ^18^F-FDG PET/CT have been well-known to reflect the increased myeloid activity resulting from systemic inflammation, thereby being useful as systemic inflammation surrogate markers [17,20]. For the evaluation of metabolic activity of spleen and BM, ROIs were placed on spleen and BM of spines (L3 to L5) from all axial slices. Mean SUVmax from these ROIs were defined as spleen SUVmax and BM SUVmax, respectively [13,17]. To assess the reliability of SUV measurements on targeted ROIs between the two nuclear medicine physicians, we performed intra- and inter-observer correlation analyses, and both correlation analyses showed excellent reliability, with a correlation coefficient > 0.9.

### 2.5. Statistical Analysis

All data were presented as mean ± standard deviation. The Pearson chi-squared (χ^2^) test or Fisher’s exact test was used for comparison of categorical variables. Shapiro–Wilk test was used to evaluate the distribution of normality. For comparison of multiple groups, one-way analysis of variance (ANOVA) with post hoc Tukey test was used for parametric analysis and Kruskal–Wallis test with post hoc Dunn’s test was used for non-parametric analysis. Spearman’s correlation coefficient, multiple linear regression analysis, receiver-operating characteristic (ROC) curve analysis, and logistic regression analysis were also performed as statistical methods. SPSS version 17.0 (SPSS Inc., Chicago, IL, USA) and MedCalc version 18.5 (MedCalc, Mariakerke, Belgium) were used for all data analysis. A *p*-value of ≤0.05 was considered statistically significant.

## 3. Results

### 3.1. Clinical Characteristics

Of the 90 participants, 65 were in the CAD group (32 in AMI and 33 in CSA), and 25 were in control group. Compared with the control group, traditional cardiovascular risk factors, such as hypertension, diabetes mellitus, dyslipidemia, and smoking were more common in CAD group. The prevalence of those risk factors was similar in both AMI and CSA groups. There were significant stepwise increases in white blood cell count, carotid artery TBR, and surrogate markers for systemic inflammation, such as hsCRP, spleen SUVmax, and BM SUVmax [17,20], from the control to the CSA and to the AMI groups (Table 1). The baseline characteristics of all study subjects are shown in Table 1.

### 3.2. SM Metabolic Activity Is Increased in CAD

**Figure 1:**
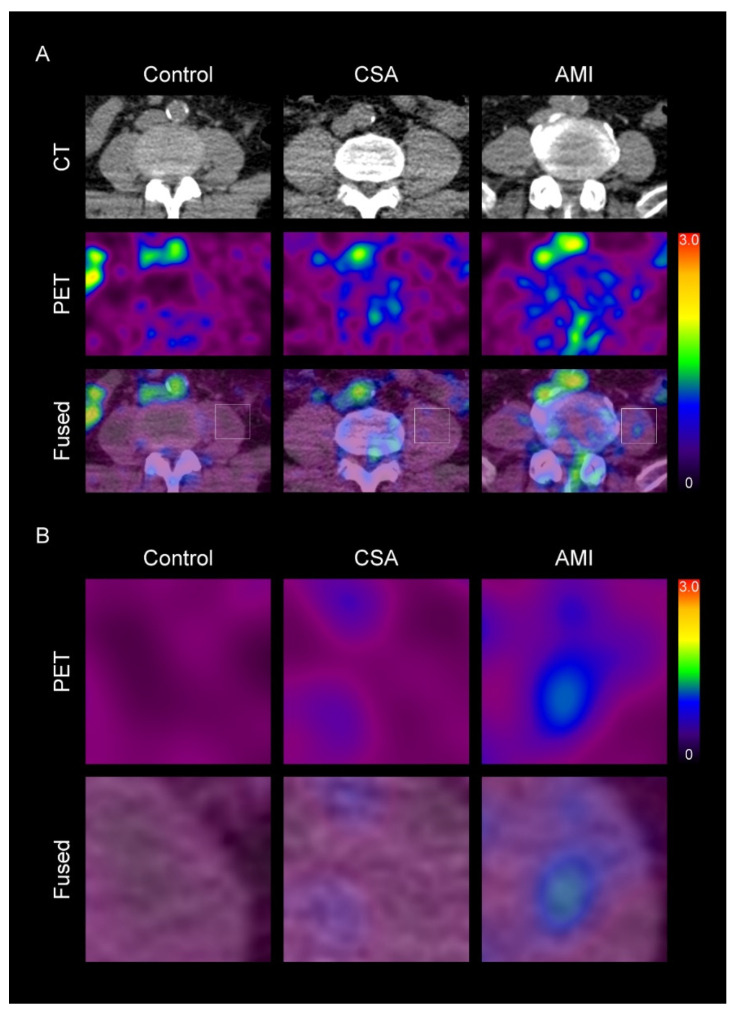
Representative images of skeletal muscle (SM) metabolic activity according to the severity of coronary artery disease (CAD) (**A**), and their corresponding magnified views (**B**). CSA, chronic stable angina; AMI, acute myocardial infarction; CT, computed tomography; PET, positron emission tomography. The levels of standardized uptake value (SUV) are shown in a color scale (high-red to low-black). In the SM region, SUV was higher in CAD group than in control group. Furthermore, in the CAD group, SUV was also higher in the AMI group than in CSA group.

**Figure 2:**
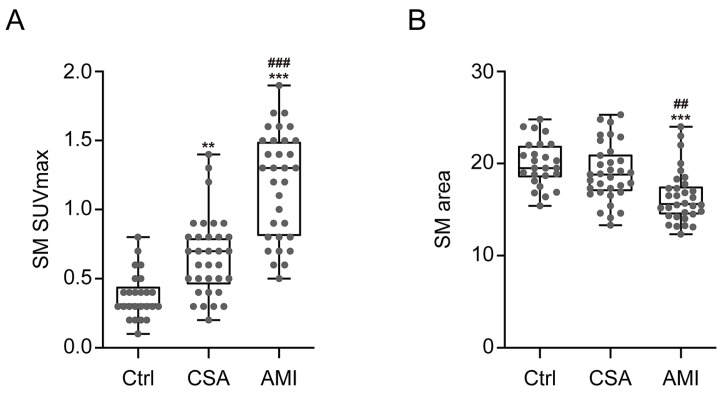
Comparison of SM SUVmax (**A**) and SM area (**B**) according to the severity of CAD. Ctrl, *n* = 25; CSA, *n* = 33; AMI, *n* = 32. Ctrl, control; SUVmax, maximum standardized uptake value. The *p*-values were determined, using one-way analysis of variance (ANOVA) with post hoc Tukey test. *** *p* < 0.001; vs. Ctrl, ** *p* < 0.01; vs. Ctrl, ^###^ *p* < 0.001; vs. CSA, ^##^ *p* < 0.01; vs. CSA.

As shown in Figure 1 and Figure 2A, SM SUVmax was highest in participants with AMI, intermediate in CSA, and lowest in the control group (1.2 ± 0.4 vs. 0.7 ± 0.3 vs. 0.4 ± 0.2, *p* < 0.001, respectively). AMI group presented significant higher SM SUVmax than the CSA and control groups (*p* < 0.001). Furthermore, CSA group also showed significant higher SM SUVmax than control group (*p* < 0.01). Regarding SM area, as shown in Figure 2B, the AMI group showed a significantly lower SM area than the CSA (16.4 ± 2.9 vs. 19 ± 3.2, *p* < 0.01) and control groups (16.4 ± 2.9 vs. 20 ± 2.5, *p* < 0.001). However, there was no significant difference between the CSA and control groups.

### 3.3. Relationship between SM Metabolic Activity and Arterial and Systemic Inflammation

SM SUVmax showed significant positive correlation with carotid artery TBR, spleen SUVmax, BM SUVmax, and hsCRP, whereas SM area showed significant negative correlation with those surrogate markers for arterial and systemic inflammation (Table 2).

Univariate regression analysis showed that carotid artery TBR was significantly associated with dyslipidemia, hsCRP, spleen SUVmax, BM SUVmax, SM SUVmax, and SM area (Table 3). A further multiple regression analysis showed that spleen SUVmax and SM SUVmax were independent contributors to carotid artery TBR (*R*^2^ = 0.469) (Table 3).

### 3.4. Comparison of SM Metabolic Activity and SM Area for the Prediction of AMI

**Figure 3:**
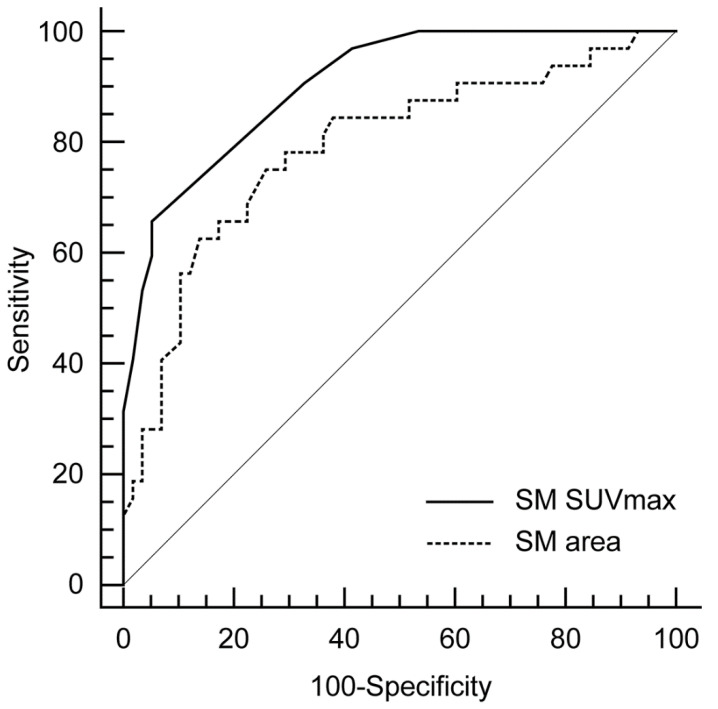
Comparison of receiver-operating characteristics (ROCs) curve analyses for the prediction of AMI.

According to the ROC curve analysis, as shown in Figure 3, the optimal cutoff value of SM SUVmax for prediction of AMI was 0.9, with a sensitivity of 65.6% and a specificity of 94.8%. Area under the curve (AUC) was 0.905 (95% confidence interval 0.824–0.956; standard error 0.03; *p* < 0.001). Considering the SM area, the optimal cutoff value of SM area for AMI was 17.3 with a sensitivity of 75% and a specificity of 74.1%. AUC was 0.79 (95% confidence interval 0.691–0.869; standard error 0.05; *p* < 0.001). Furthermore, SM SUVmax had a significant higher AUC than SM area (*p* = 0.05, Figure 3).

Univariate and multivariate logistic regression analyses showed that dyslipidemia, SM SUVmax, and SM area were significantly associated with AMI (Table 4). Interestingly, among the included variables, SM SUVmax had the highest odds ratio for AMI.

## 4. Discussion

To the best of our knowledge, this is the first prospective study reporting the relationship between SM metabolic activity and the severity of CAD, including AMI, by using ^18^F-FDG PET/CT. In this study, we clearly identified that SM metabolic activity defined as SM SUVmax was highest in patients with AMI, intermediate in CSA, and lowest in the control group. Furthermore, SM SUVmax was significantly associated with arterial and systemic inflammation and could predict AMI.

Myocytes, adipocytes, and inflammatory cells are the predominant cell populations in inflamed SM [6]. Growing evidences suggest that increased immune cell infiltration in SM is the hallmark of inflamed SM and may constitute the major inflammatory cells in SM [14,21,22,23]. During inflammation, glucose uptake is upregulated in infiltrated inflammatory cells, such as macrophages [24,25]. Macrophages use insulin-independent glucose transporter-1 (GLUT-1) for glucose uptake and, thus, do not develop insulin resistance in the inflammatory process [25,26]. In contrast, both myocytes and adipocytes express insulin-dependent GLUT-4 for glucose uptake [4,6,8,27]. During inflammation, macrophages secrete pro-inflammatory cytokines, such as TNF-α and interleukin-6 (Il6), thereby downregulating GLUT-4 in both myocytes and adipocytes, leading to a decrease glucose uptake of those cells in inflamed SM, which is also involved with the development of insulin resistance [24,28,29]. Thus, we suspect that SM SUVmax could indicate the maximal inflammatory metabolic activity of inflammatory cells, such as macrophages in SM.

Inflamed SM could contribute to the development of systemic inflammation and remote arterial inflammation via secretion of pro-inflammatory cytokines into systemic circulation [4,5,6]. Several previous in vivo animal studies have been reported that CAD induces increased level of circulating monocytes, thereby promoting infiltration of macrophages in remote atherosclerotic plaque, which could result in another subsequent CAD event such as recurrent MI [30,31,32]. This finding is further supported by recent clinical studies using ^18^F-FDG PET/CT that both systemic and carotid artery inflammation are upregulated according to the severity of CAD and related with each other [17,20,33], which are also consistent with results from present study.

Therefore, next, we explored the relationship between SM metabolic activity and systemic and remote arterial inflammation in CAD patients. In the present study, we found that SM metabolic activity, defined as SM SUVmax, was positively correlated with systemic inflammation and independently associated with carotid artery inflammation. Furthermore, it was a powerful predictor of AMI. Thus, collectively, these findings suggest that SM SUVmax can reflect the inflammatory burden of SM, which may affect the remote vasculature that eventually contributes to escalation of CVD risk.

Inflamed SM also contributes to the loss of muscle mass, which is referred to as sarcopenia [34]. Regarding SM area, the measurement of the cross-sectional area of the psoas muscle is known to reflect the total body skeletal muscle mass [35]. Thus, SM area could be used as a surrogate marker for total skeletal muscle mass. Several previous observational studies have been reported that reduced skeletal muscle mass are significantly associated with CAD [36,37,38], which was also consistent with our results. However, in contrast to SM SUVmax, SM area was not independently associated with carotid artery inflammation (Table 3). Furthermore, it did not show superior predictability for AMI than SM SUVmax (Figure 3 and Table 4). Thus, we believe that SM SUVmax evaluated by ^18^F-FDG PET/CT would be more suitable to evaluate the relationship between inflamed SM and the risk of CAD than SM area.

Recently, SM inflammation has been regarded as a promising target for treatment of CVD [6]. In addition, several previous studies report that physical and pharmacological therapies could reduce the SM inflammation [39,40]. Thus, in this point of view, assessment of SM inflammation is crucial for risk stratification and monitoring treatment response in patients with CVD. Although SM biopsy can be considered as a gold standard to evaluate SM inflammation, it is a cumbersome and invasive procedure in actual clinical practice. Thus, SM SUVmax evaluated by ^18^F-FDG PET/CT could be used as a readily measurable, non-invasive surrogate marker for reflecting the SM inflammation.

This study has several limitations. First, although this was a prospective study, the present study was conducted at a single institute, with a small-sized sample and cross-sectional design, which can induce selection bias. A further large-population study is warranted, to confirm our findings. Second, unlike to the AMI and CSA group, we could not perform the coronary angiography in the control group, to evaluate the extent of coronary atherosclerosis. Third, we were unable to perform SM biopsy to acquire tissue samples from SM that could support our findings. Fourth, we were unable to control all the possible factors that could affect ^18^F-FDG distribution, including glucose and plasma insulin levels, nor the image taking time after ^18^F-FDG injection. Finally, physical activity level is known to have an effect on inflammatory status of SM [39]. However, we could not control the physical activity level of study participants, which might affect the result of this study.

## 5. Conclusions

Taken together, we provide strong evidence that SM metabolic activity, defined as SM SUVmax and evaluated by ^18^F-FDG PET/CT, was associated with coronary plaque instability and synchronized with the systemic and arterial inflammation, which may lead to another future adverse CVD event. Furthermore, it showed superior predictability for the prediction of AMI. Collectively, these findings provide additional evidence for SM SUVmax as an imaging biomarker for CVD risk and offer insights into exploring the interplay between SM metabolic activity and coronary plaque instability.

## Figures and Tables

**Table 1:** Baseline characteristics of participants.

	Control, *n* = 25	CSA, *n* = 33	AMI, *n* = 32	*p*
Age, y	57.1 ± 7.8	61.2 ± 11.5	57 ± 11.6	0.206
Men, *n* (%)	6 (24)	24 (72.7) *	21 (65.6) ^†^	<0.001
BMI, kg/m^2^	23.5 ± 2.9	26 ± 4 *	24.6 ± 2.6	0.021
WC, cm	80.9 ± 7.5	92.3 ± 11.4 *	83.4 ± 16.3 ^‡^	<0.001
Hypertension, (%)	1 (4)	19 (57.6) *	15 (46.9) ^†^	<0.001
DM (%)	2 (8)	13 (39.4) *	13 (40.6) ^†^	0.021
Dyslipidemia (%)	2 (8)	16 (48.5) *	19 (59.4) ^†^	<0.001
Current Smokers, n (%)	2 (8)	13 (39.4) *	13 (40.6) ^†^	0.021
Statin Use (%)	0	11 (33.3)	9 (28.1)	0.649
Total Cholesterol, mg/dL	189 ± 25.3	156.4 ± 35.2 *	186.9 ± 43.6 ^‡^	0.001
Triglycerides, mg/dL	86.7 ± 44.3	160.4 ± 99.8 *	136.6 ± 142.1 ^†‡^	<0.001
HDL Cholesterol, mg/dL	59.4 ± 15.7	48.7 ± 15.2 *	45 ± 11.8 ^†^	0.001
LDL Cholesterol, mg/dL	115.3 ± 24.1	91.9 ± 30.1 *	124.3 ± 41.7 ^‡^	<0.001
HbA1c, %	5.7 ± 0.4	7 ± 1.6 *	6.9 ± 2.1 ^†^	<0.001
WBC, ×10^3^/μL	5 ± 1.3	6.5 ± 1.2 *	10.9 ± 3.3 ^†‡^	<0.001
hsCRP, mg/L	0.6 ± 0.6	1.5 ± 1.6 *	3.5 ± 3.1 ^†‡^	<0.001
VAT Area, cm^2^	147 ± 57.1	261.3 ± 110.6 *	209.1 ± 80.1 ^†^	<0.001
Peak CK-MB, ng/mL	…	…	145.6 ± 127.3	…
Peak Troponin-T, ng/mL	…	…	3.7 ± 4.6	…
Metabolic Parameters				
Carotid Artery TBR	1.2 ± 0.1	1.4 ± 0.4 *	2.1 ± 0.4 ^†‡^	<0.001
Spleen SUVmax	1.5 ± 0.3	2 ± 0.3 *	2.6 ± 0.4 ^†‡^	<0.001
BM SUVmax	0.8 ± 0.4	1.2 ± 0.6 *	1.7 ± 0.2 ^†‡^	<0.001

All data were presented as mean ± standard deviation or *n* (%). The *p*-values were determined using ANOVA with post hoc Tukey test or Kruskal–Wallis test with post-hoc Dunn’s test for continuous variables and Pearson chi-squared (χ^2^) test or Fisher exact test for categorical variables. * *p* < 0.05, Control vs. CSA, ^†^ *p* < 0.05, Control vs. AMI, ^‡^ *p* < 0.05, CSA vs. AMI. CSA, chronic stable angina; AMI, acute myocardial infarction; BMI, body mass index; WC, waist circumference; DM, diabetes mellitus; HDL, high-density lipoprotein; LDL, low-density lipoprotein; HbA1c, hemoglobin A1c; WBC, white blood cell; hsCRP, high-sensitivity C-reactive protein; VAT, visceral adipose tissue; CK-MB, creatine kinase-MB; TBR, target-to-background ratio; SUVmax, maximum standardized uptake value; and BM, bone marrow.

**Table 2:** Spearman correlation analysis between SM SUVmax, SM area, and systemic and arterial inflammation parameters.

	SM SUVmax		SM Area	
	*r*	*p*	*r*	*p*
Carotid Artery TBR	0.599	<0.001 *	−0.341	0.001 *
Spleen SUVmax	0.581	<0.001 *	−0.428	<0.001 *
BM SUVmax	0.539	<0.001 *	−0.432	<0.001 *
hsCRP	0.546	<0.001 *	−0.295	0.006 *

Data were correlation coefficients from correlation analysis. SM, skeletal muscle; SUVmax, maximum standardized uptake value; TBR, target-to-background ratio; BM, bone marrow; hsCRP, high-sensitivity C-reactive protein. * Statistically significant difference.

**Table 3:** Univariate and multivariate analyses for carotid artery TBR values.

	Univariate		Multivariate	
Variable	Coefficients (95% CI)	*p*	Coefficients (95% CI)	*p*
Age	−0.004 (−0.017–0.009)	0.553		
Sex	0.253 (−0.029–0.535)	0.078		
BMI	−0.01 (−0.053–0.033)	0.657		
WC	−0.006 (−0.016–0.005)	0.318		
HTN	0.026 (−0.272–0.324)	0.862		
DM	0.28 (−0.026–0.585)	0.072		
Dyslipidemia	0.33 (0.044–0.615)	0.024 *	0.073 (−0.181–0.326)	0.571
Current Smokers	0.1 (−0.215–0.416)	0.53		
hsCRP	0.01 (0.003–0.018)	0.008 *	0.004 (−0.003–0.01)	0.249
Spleen SUVmax	0.744 (0.526–0.962)	<0.001 *	0.424 (0.139–0.708)	0.004 *
BM SUVmax	0.438 (0.271–0.606)	<0.001 *	0.03 (−0.198–0.258)	0.793
SM SUVmax	0.892 (1.225–1.684)	<0.001 *	0.545 (0.236–0.855)	0.001 *
SM area	−0.072 (−0.114–−0.031)	0.001 *	−0.004 (−0.045–0.037)	0.843

BMI, body mass index; WC, waist circumference; HTN, hypertension; DM, diabetes mellitus; hsCRP, high-sensitivity C-reactive protein; SUVmax, maximum standardized uptake value; BM, bone marrow; SM, skeletal muscle; and CI, confidence interval. * Statistically significant difference.

**Table 4:** Univariate and multivariate analyses for prediction of acute myocardial infarction.

	Univariate		Multivariate	
Variable	OR (95% CI)	*p*	OR (95% CI)	*p*
Age (Continuous)	0.978 (0.939–1.019)	0.978		
Sex (Female vs. Male)	1.782 (0.73–4.352)	0.205		
BMI (Continuous)	0.974 (0.853–1.111)	0.692		
WC (Continuous)	0.976 (0.941–1.012)	0.191		
HTN (Negative vs. Positive)	0.541 (0.707–4.175)	0.232		
DM (Negative vs. Positive)	1.87 (0.745–4.696)	0.183		
Dyslipidemia (Negative vs. Positive)	3.085 (1.253–7.598)	0.014 *	16.197 (1.8–145.765)	0.013 *
Current Smokers (None vs. Yes)	1.733 (0.688–4.369)	0.244		
SM SUVmax (≤0.9 vs. >0.9)	55 (13.416–225.475)	<0.001 *	139.317 (12.843–1511.283)	<0.001 *
SM Area (>17.3 vs. ≤17.3)	15.48 (5.047–47.478)	<0.001 *	8.965 (1.913–42.012)	0.005 *

BMI, body mass index; WC, waist circumference; HTN, hypertension; DM, diabetes mellitus; SM, skeletal muscle; SUVmax, maximum standardized uptake value; OR, odds ratio; and CI, confidence interval. * Statistically significant difference.

## Data Availability

The data presented in this study are available on request from the corresponding author.
